# Editorial: Plant mineral microbe interactions, vol II

**DOI:** 10.3389/fmicb.2026.1903162

**Published:** 2026-07-28

**Authors:** Muhammad Zahid Mumtaz, Brahim Bouizgarne, Tereza Cristina Luque Castellane, Elena Tamburini

**Affiliations:** 1 Institute of Molecular Biology and Biotechnology, The University of Lahore, Lahore, Pakistan; 2 College of Agronomy, Gansu Agricultural University, Lanzhou city, China; 3 Laboratory of Plant Biotechnology (BiotecV), Faculty of Sciences, Ibn Zohr University, Agadir, Morocco; 4 Instituto de Pesquisa em Bioenergia, IPBEN, Jaboticabal, São Paulo, Brasil; 5 Department of Biomedical Sciences, University of Cagliari, Monserrato city, Italy

**Keywords:** biogeochemical cycling, microbial mineral transformation, mineral weathering and dissolution, plant microbe interactions, plant mineral microbe interactions, rhizosphere microbiome, soil microbiome

Building upon the foundational insights of Volume I, Plant Mineral Microbe Interactions, Vol II expands the scientific scope by shifting focus from basic symbiotic pathways toward the precise mechanistic modeling and functional integration of microbial communities. While Vol I established the core dynamics of rhizosphere isolation, this second volume delivers a more comprehensive cohort of peer-reviewed articles targeting multi-strain synthetic consortia (SynComs) and engineered microbiome responses under extreme climate stress.

Plant Mineral Microbe Interactions, driven by a surge in high-quality global submissions, this second edition accommodates a cohort of 17 peer-reviewed articles, demonstrating the rapid global growth and interest in the discipline of this emerging field. These studies, authored by scientists worldwide, provide mechanistic and applied insights into microbial ecology, microbial networks and dynamics, plant growth promotion, pathogen suppression, soil fertility, nutrient cycling, soil carbon dynamics and crop health, and resilience to climate change of managed and natural ecosystems. Plant Mineral Microbe Interactions, Vol II advances the transition from descriptive ecology to a mechanistic understanding of rhizosphere function. These interactions provide a foundation for next-generation approaches to sustainable nutrient management and agroecosystem resilience under abiotic stresses by elucidating the coupling between biological and geochemical processes. The findings published in this Research Topic revealed an emerging paradigm of an interconnected and self-organized rhizospheric system in which microbial metabolism, mineral transformation, and plant physiological responses jointly regulate nutrient dynamics. Understanding these interconnected processes amid global soil degradation, climate change, and biodiversity loss is crucial for sustainable food production.

The rhizosphere zone dynamically regulated reaction network where plants, microbes, and minerals co-govern the flux, speciation, and bioavailability of nutrients (York et al., 2016). The contributions compiled in this Research Topic collectively advance a mechanistic framework of the rhizosphere as a tightly coupled physicochemical-biological system being regulated by feedback of microbial metabolism, root exudates, and mineral weathering. Root exudates, composed of low-molecular-weight organic acids to complex secondary metabolites, are the sources of microbial substrates and act as geochemical agents that alter soil pH, chelate metal ions, and increase mineral dissolution (Ma et al., 2022). This process is further modulated by microbial communities that selectively amplify mineral dissolution through diverse biochemical mechanisms. Mineral-dissolving microorganisms produce organic acids and enzymes that transform insoluble minerals into plant-available forms. These microbes catalyze redox transformations and alter mineral crystallinity and surface reactivity and release bioavailable nutrients in elemental or organic complexed forms (Natarajan, 2018). These redox processes are associated with plant signaling pathways as plants respond to nutritional deficiency by modulating exudate composition and altering microbial community functions. It creates a feedback system in which plant nutritional status acts as a key regulator of the consequences of microbially mediated mineral dynamics (Pradhan et al., 2025). This mechanism is further controlled by extracellular polymeric substances, which act as physicochemical scaffolds and retain water, chelate metal ions, buffer pH fluctuations, and stabilize transient intermediates to modulate the kinetics of mineral dissolution and precipitation (Husen, 2024). The various microbial secondary metabolites, including siderophores, phenazines, flavins, quinones, hydroquinone, melanin, and humic-like substances, act as molecular agents that directly interact with plant roots and mineral surfaces. Their function extends beyond nutrient acquisition to competitive interactions within microbial communities and circuitously affects plant uptake pathways. These redox-active compounds mediate electron transfer to decouple microbial metabolism from immediate contact with plant roots and mineral surfaces (Anas et al., 2026).

Plant mineral microbe interaction is controlled by genetic and regulatory mechanisms. Microbial genomes encoding suites of transporters and regulatory networks are involved in response to plant and mineral-derived signals. Quorum-sensing regulatory systems empower microbes to synchronize biofilm formation, exoenzyme production, and related collective behaviors. The transporter families and transcriptional regulators in plants involved in nutrient uptake progressively responded to microbial metabolites (Mohan et al., 2019). These mechanisms suggest a bidirectional cross-talk that operates alongside material fluxes. The studies published in this Research Topic provide novel insight into the emerging concept of a mineral-selective microbiome rather than the plant-driven microbial community composition, as mineralogy itself acts as a selective pressure. Clay minerals and their associated ions differ in surface chemistry and reactivity to shape microbial communities, their metabolic strategies, and mineral transformation processes in the rhizosphere. Such mineralogical filtering interacts with plant and environmental conditions to shape context-dependent microbiomes with distinct functional capacities (Zhang et al., 2026). The above mechanistic understandings open avenues for engineering rhizosphere functions. The applied strategies of targeted inoculation with mineral-solubilizing microbes, altering root exudate through biotechnological approaches, and soil amendment with specific minerals can be used to enhance nutrient use efficiency and stress resilience. Yet, the inherent complexity and context-dependent plant mineral microbe interactions preclude overly simplistic interpretations, thereby specifying the various feedback systems through which local perturbations can propagate across the system.

The mineral microbe interactions play a pivotal role in affecting soil fertility and crop health. Two published works attempted to unlock the plant growth promotion potential of *Bacillus* species through nutrient availability. Hussain et al. revealed that mineral-solubilizing Gram-positive *Bacillus* increased wheat growth and photosynthesis through their metabolic potential of siderophore production, exopolysaccharide, and urease activity, leading to improved nutrient availability and physiological traits. Xu et al. applied cellulolytic *Bacillus cereus* B9 combined with an organic amendment and reported increased nutrient availability and litter decomposition in soil. This strain possesses endoglucanase and β-glucosidase encoding genes, which perform cellulase activity and increase soil ammonium through the dissimilatory nitrate reduction pathway. Its inoculation along with vermicompost showed higher nitrogen and carbon metabolic activity, organic matter, and nutrient turnover. Another work by Chaves et al. focused on polyhydroxybutyrate (PHB), an intracellular carbon storage polymer produced by PGPRs in soil and involved in plant-microbe interactions, particularly in root colonization and adaptation during stress phases (nutrient limitation, drought, UV, etc.). Under nitrogen-limited conditions, authors found coordinated regulation of genes involved in polyhydroxybutyrate (PHB) metabolism in the wild-type of *Herbaspirillum rubrisubalbicans* M1, resulting in higher PHB storage and efficient *Sorghum* root colonization. However, depletion of the *Hrubri_0242* function altered the regulation of these genes and the mutant resulted in reduced PHB production, motility, biofilm formation, adhesion, and efficiency in *Sorghum* colonization and impaired carbon storage under the same nitrogen limitations.

Besides bacteria, fungi also show plant growth-promoting effects, mainly through enhancing nutrient availability. The ecological and functional versatility of endophytic fungi is reviewed by Du et al., who highlighted the complex plant–fungal molecular interaction mechanisms by which these endophytic fungi promote plant growth through increasing nutrient acquisition, phytohormone-mediated regulation, and activation of stress-response antioxidant and osmotic pathways. Cai et al. reported the dual function of *Penicillium korosum* and *Penicillium aculeatum* isolated from the rhizosphere of wild *Fritillaria thunbergii* in terms of disease suppression and plant growth promotion by solubilizing phosphorus, producing siderophores, antagonizing pathogens, and upregulating defense-related genes.

Nowadays, research has also gained interest in functional resilience of beneficial microorganisms to harsh environmental conditions, particularly to drought. However, adaptive strategies seem to be developed by harmful or undesirable microorganisms. Philipp et al. demonstrated increase in pathogenic fungi in intensively managed grasslands in extreme drought conditions context while Søchting et al. identified a drought stress tolerant algal species of *Apatococcus (A. ammoniophilus)*, which represents a nuisance to conifers, including Christmas trees, leading to economic losses. This species accumulates low molecular weight carbohydrates (e.g., erythritol, arabitol, and trehalose) and a unique UV-absorbing mycosporine-like amino acid (MAA), which protects against desiccation and UV radiation, helping this algal species to adapt to aeroterrestrial environments.

Nine works focused on factors influencing microbial community dynamics, largely linked to soil properties and plant growth potential. Some of these works focused on bacterial and/or fungal dynamics, including beneficial and pathogenic ones. These parameters are: **(i)** *Nutrient availability*: Quijia-Pillajo et al. observed a nitrogen fertilization rate of 200 mg L^−1^ as a key driver of bacteriome dynamics across the substrate-plant continuum in Petunia × hybrida. This nitrogen input restructured the bacterial community composition while reducing bacterial diversity across the soilless substrate, rhizosphere, and endosphere. (**ii**) *Soil properties*: Zhang Q. et al. investigated arbuscular mycorrhizal fungi in rhizosphere soil and roots of Cabernet Sauvignon grapevines from seven ecological regions at the eastern foot of the Helan Mountains in Ningxia, China. They found spatial heterogeneity in colonization and spore densities of arbuscular mycorrhizal fungi. The grape rhizosphere in this region showed rich diversity of arbuscular mycorrhizal fungi, with dominance by *Glomus* and *Glomus melanosporum* influenced by soil pH and organic matter. (iii) *Cultural and agricultural practices* are of major importance in regulating microbial dynamics in soils. Li X. et al. reported that film-straw dual mulching increased tomato yield, dry matter accumulation, and root growth through the selective abundance of plant growth-promoting microorganisms associated with nutrient cycling. Whereas, straw mulching, despite increasing nutrient availability, resulted in lower yield due to a higher abundance of pathogenic fungi. Li L. et al. reported that continuous cropping of *Platycodon grandiflorus* causes soil acidification and root rot disease due to higher abundance of acid-tolerant pathotrophic and saprotrophic fungi and a reduction in the abundance of beneficial fungal species. Zhang Y. et al. also reported soil acidification and a reduction in organic matter and nutrient availability due to the continuous cultivation of lilies. It leads to a reduction in organic matter-degrading and nutrient-cycling microorganisms (e.g., *Proteobacteria, Sphingomonas, Ascomycota*, and *Basidomycota*) while showing a higher abundance of *Acidobacteriota* and *Fusarium*.

(iv) *Land-use history*: the land-use and cultivation history regulate plant mineral and microbe interactions by restructuring soil physicochemical properties, nutrients and microbial communities. Chen et al. studied the transition from native soil to alfalfa-cultivated soil in alpine desert lands and revealed a shift in the soil microbial community, with higher abundances of plant growth-promoting bacteria (e.g., *Arthrobacter* and *Pseudomonas*) and reduced abundances of decomposers. Long-term alfalfa cultivation improved soil quality by progressively homogenizing microbial diversity driven by soil pH and organic matter. Philipp et al. demonstrated that intensively managed grasslands are more vulnerable to extreme drought conditions with increase in fungi of pathogenic potential, while less managed grasslands were less sensitive to drought. The fungal communities were more responsive to drought stress than the bacterial community, particularly in croplands with higher carbon cycling enzymatic activities. Sun et al. investigated the changes in soil nutrients and bacterial community in undisturbed grassland and reclaimed cropland. Undisturbed grassland showed higher retention of ammonium, nitrate, and total phosphorus compared to reclaimed cropland. The reclaimed cropland showed reduced interspecies interactions and network complexity, with increased stability in bacterial community composition. Gao et al. reported that extreme vegetation disturbance reduces bacterial abundance and fungal diversity by limiting soil moisture and nutrient availability, while soil pH, moisture, and nitrate concentration appeared as primary drivers of microbial abundance and diversity under extreme disturbance.

Furthermore, forest ecosystems are well represented in this Research Topic, mainly in studies addressing soil carbon stabilization and microbial community dynamics. Indeed, microbial networks and substrate properties were also reported as influenced by forest type and silvicultural methods. Tan et al. reported that soil organic carbon sequestration was jointly controlled by iron oxides and microbial community, with a non-linear pattern across forest age that peaks around 20 years and declines thereafter. Broadleaf forests enhanced enzymatically mediated iron-bound organic carbon coprecipitation, while mixed forests showed synergistic interactions between acidophilic bacteria and iron oxides, which contribute to carbon sequestration in deeper soils of mature forests. Liu et al. reported the higher impact of the uneven-aged tree mixing mode on microbial alpha diversity compared to the even-aged plantation mixing mode. Mixed plantations of even- and uneven-aged plantations showed higher complexity in fungal co-occurrence networks and harbored more bacterial and fungal keystone species. Fungal communities were more sensitive to mixed plantations, while bacterial communities were regulated by soil pH.
